# A Primary Physiological Role of Toxin/Antitoxin Systems Is Phage Inhibition

**DOI:** 10.3389/fmicb.2020.01895

**Published:** 2020-08-13

**Authors:** Sooyeon Song, Thomas K. Wood

**Affiliations:** ^1^Department of Animal Science, Jeonbuk National University, Jeonju-si, South Korea; ^2^Department of Chemical Engineering, Pennsylvania State University, University Park, PA, United States

**Keywords:** toxin/antitoxin systems, phage, CRISPR-Cas, toxin–antitoxin system, plasmid

## Abstract

Toxin/antitoxin (TA) systems are present in most prokaryote genomes. Toxins are almost exclusively proteins that reduce metabolism (but do not cause cell death), and antitoxins are either RNA or proteins that counteract the toxin or the RNA that encodes it. Although TA systems clearly stabilize mobile genetic elements, after four decades of research, the physiological roles of chromosomal TA systems are less clear. For example, recent reports have challenged the notion of TA systems as stress-response elements, including a role in creating the dormant state known as persistence. Here, we present evidence that a primary physiological role of chromosomally encoded TA systems is phage inhibition, a role that is also played by some plasmid-based TA systems. This includes results that show some CRISPR-Cas system elements are derived from TA systems and that some CRISPR-Cas systems mimic the host growth inhibition invoked by TA systems to inhibit phage propagation.

## Toxin/Antitoxin System Overview

Chromosomal toxin/antitoxin (TA) systems are prevalent in Bacteria and Archaea (Yamaguchi et al., 2011), and bacteria often have multiple members. For example, *Escherichia coli* K-12 has at least 39 TA systems (Kim and Wood, 2016). However, their role in cell physiology is disputed, even though it is highly unlikely they are merely addiction modules given their prevalence in most genomes and their redundancy (Sberro et al., 2013). For this review, the phrase “chromosomal TA systems” excludes horizontally acquired genomic islands and active temperate phages but includes cryptic (inactive) prophages because they have been integrated into the host genome (Wang et al., 2010).

TA systems are encoded by adjacent genes, usually consisting of two components and usually result in the activation of a toxin that reduces metabolism. Contrary to much of the literature, toxins are probably not activated by specific degradation of bound antitoxins, which are structured and thereby not likely substrates of proteases such as Lon, but, instead, toxins are probably activated by *de novo* RNA synthesis (Song and Wood, 2020c). Toxins reduce metabolism in diverse ways, for example, by reducing ATP production by damaging the cell membrane (Cheng et al., 2014), by inhibiting translation through mRNA/tRNA/rRNA modifications (Winther et al., 2016; Culviner and Laub, 2018), and by impeding replication through adenylylation of DNA gyrase and topoisomerase IV (Harms et al., 2015).

TA systems are mobile through horizontal gene transfer (Ramisetty and Santhosh, 2015), are often autoregulated (Page and Peti, 2016), and may be arranged in cascades (Wang et al., 2013). There are eight classification groups for TA systems on the basis of the antitoxin mechanism (Song and Wood, 2020c): (i) antitoxin anti-sense RNA inhibits toxin mRNA in type I systems (Gerdes et al., 1986a); (ii) antitoxin protein binds and inhibits toxin protein in type II systems (Ogura and Hiraga, 1983); (iii) antitoxin RNA binds and inhibits the protein toxin in type III systems (Fineran et al., 2009); (iv) antitoxin protein prevents the protein toxin from binding its target in type IV systems (Masuda et al., 2012); (v) antitoxin enzyme, an RNase, degrades specifically toxin mRNA in type V systems (Wang et al., 2012); (vi) antitoxin protein stimulates the degradation of toxin protein in type VI systems (Aakre et al., 2013); (vii) antitoxin enzyme oxidizes a cysteine residue of the protein toxin to inactivate it in type VII systems (Marimon et al., 2016); and (viii) antitoxin RNA inactivates the toxin RNA by anti-sense binding, but the toxin functions as a small RNA rather than as a protein in type VIII systems (Choi et al., 2018).

## Toxin/Antitoxin Systems Stabilize Mobile Genetic Elements

In contrast to chromosomally encoded TA systems, the physiological role of TA systems is more clear for mobile genetic elements like plasmids. The stabilization role was established with the discovery of TA systems via the report of the CcdB/CcdA type II TA system, which stabilizes the mini-F plasmid (Ogura and Hiraga, 1983). Subsequently, the type I TA system, Hok/Sok, was shown to stabilize the R1 plasmid (Gerdes et al., 1986b). Since these initial reports with plasmid stabilization, many examples of TA systems stabilizing plasmids have been documented. Furthermore, the integrative and conjugative element SXT in *Vibrio cholerae* has been shown to be stabilized via the MosT/MosA TA system (Wozniak and Waldor, 2009), and TA systems stabilize prophages (Soutourina, 2019). Hence, although not all TA systems stabilize plasmids, the physiological role of TA systems for the stabilization of non-chromosomal mobile genetic elements is clear.

## Toxin/Antitoxin Systems and the General Stress Response

Unlike the role of TA systems in stabilizing mobile genetic systems and in phage inhibition, the physiological role of chromosomal TA systems for stress resistance is being challenged. For example, the Van Melderen group published a series of negative results in which they claimed the chromosomal *Escherichia coli* MqsR/MqsA TA system had no role in stress resistance, on the basis of a lack of transcription response and lack of phenotype upon deleting *mqsRA* (Fraikin et al., 2019). Critically, their transcription results were invalidated within a few months in that *mqsRA* transcription was shown to increase by over 181-fold during amino acid stress and 90-fold during oxidative stress (LeRoux et al., 2020b). In addition, although the Van Melderen group did not find a phenotype upon deleting *mqsRA*, including in the presence of bile acid stress (Fraikin et al., 2019), the Gross lab reported in *Cell* that *E. coli mqsR* has reduced growth with fusidic acid and radicicol, and they reported a hypomorphic *mqsA* mutation (i.e., a strain with reduced MqsA because *mqsA* is lethal owing to extreme MqsR toxicity; Brown et al., 2009) has reduced growth with the bile acid deoxycholate (Nichols et al., 2011). Furthermore, we showed clearly that deleting *mqsRA* alters the cell’s response to bile acid in seven independent experiments with 2 to 5% deoxycholate and identified the importance of periplasmic protein YgiS as responsible for deoxycholate uptake (Kwan et al., 2015). In addition, we discovered that antitoxin MqsA binds and represses the promoter of the master regulator of the stress response, sigma factor RpoS, through the palindrome that MqsA uses for its own transcription regulation (Wang et al., 2011); deletion of *mqsRA* along with five other TA systems increased both hydrogen peroxide and acid resistance by a factor of 10, and deletion of *mqsRA* increased biofilm formation, c-di-GMP levels, cellulose/curli (Wang et al., 2011). Also, three separate groups have found that MqsR/MqsA is related to antibiotic tolerance based upon deletion of *mqsR* (Kim and Wood, 2010; Luidalepp et al., 2011; Wu et al., 2015). Convincingly, these results are based on deletions, rather than production of the TA module from plasmids.

In addition, other phenotypes have been reported that are related to the MqsR/MqsA TA system in *E. coli*, including those related to heat shock (Richmond et al., 1999), biofilm formation (Shah et al., 2006), nitrogen starvation (Figueira et al., 2015), and nitric oxide (Partridge et al., 2009). Also, there are reported phenotypes related to the MqsR/MqsA TA system in non-*E. coli* systems including copper stress (Merfa et al., 2016), vesicles (Santiago et al., 2016), and biofilm formation (Lee et al., 2014) in *Xylella fastidiosa*. Moreover, MqsR/MqsA affects biofilm formation in *Pseudomonas fluorescens* (Wang et al., 2019) and persistence and biofilm formation in *Pseudomonas putida* (Sun et al., 2017). Similarly, other TA systems such as the MazE/MazF (Kolodkin-Gal et al., 2007), RelE/RelB (Christensen et al., 2001), and YafQ/DinJ (Zhao et al., 2016) TA systems have been linked to the general stress response; but their roles have also been disputed in *E. coli* (LeRoux et al., 2020a).

In contrast to work showing chromosomal TA systems like MqsR/MqsA affect the stress response, the impact of TA systems on persistence is not convincing, primarily because the fold changes in most persister experiments with individual TA systems are usually small (less than 10-fold) and strains with multiple TA systems deleted do not show consistent phenotypes. Persistence is an extreme stress response that occurs when a subpopulation of cells becomes dormant due to ribosome dimerization as a direct result of increased ppGpp levels (Kim and Wood, 2016; Kim et al., 2018; Song and Wood, 2020a, b; Yamasaki et al., 2020). Specifically, inactivation of 10 TA systems did not affect *E. coli* persistence for several groups (Harms et al., 2017; Goormaghtigh et al., 2018; Svenningsen et al., 2019). Similarly, deletion of 12 TA systems in *Salmonella enterica* had no effect on persistence (Pontes and Groisman, 2019).

In addition, there is little evidence that cells resuscitate by inactivating TA system toxins. For example, some have indicated (Dewachter et al., 2019) that the peptidyl-tRNA hydrolase Pth counteracts toxin TacT in *Salmonella* Typhimurium during resuscitation; however, there are no data showing that Pth plays a role in persister resuscitation (Cheverton et al., 2016). Similarly, it was reported that deactivation of HokB toxin in *E. coli* causes persister cell resuscitation; however, single-cell observations were not used (Wilmaerts et al., 2019), the experiments rely on non-physiological levels of toxin from overproduction studies (Wilmaerts et al., 2018, 2019), deleting *hokB* has no effect on persistence (Verstraeten et al., 2015), and GTPase Obg, the enzyme used to claim originally that HokB is related to persistence, reduces persistence without HokB (Verstraeten et al., 2015). Therefore, although TA systems appear to be utilized by cells to respond to stress, they are probably not utilized to form or resuscitate persister cells.

## Discovery of Toxin/Antitoxin Systems and Phage Inhibition

Restriction/modification systems are utilized to ward off phage infection; however, they also stabilize plasmids (Kulakauskas et al., 1995). Because TA systems stabilize plasmids (Ogura and Hiraga, 1983), we reasoned by the transitive property that TA systems may also inhibit phage (Pecota and Wood, 1996). In addition, we realized that temperature shock, amino acid deprivation, antibiotics, and, critically, phage infection, would alter transcription and would perhaps activate toxins of type I TA systems that rely on antisense antitoxin RNA production (Pecota and Wood, 1996); hence, we hypothesized that TA systems may be used to inhibit phage. To test this hypothesis, we induced the type I TA system Hok/Sok from the R1 plasmid and challenged with T1, T4, T5, T7, and λ phage and found T4 phage were substantially inhibited: plating efficiency was reduced by 42%, plaque size was reduced by 85%, burst size was reduced by 40%, maturation time was increased by 36%, and the latent period was increased from 30 to 60 min. The likely mechanism is that upon phage infection, T4 phage blocks host transcription in 3–4 min, which leads to elimination of Hok and Sok RNA production; the Sok RNA is then preferentially degraded, and Hok toxin is produced (Pecota and Wood, 1996). Therefore, a TA system was shown to inhibit phage. We also reasoned that phage inhibition by TA systems would be important for biofilms where cells in outer layers could protect kin (Pecota and Wood, 1996).

## Paradigm of Phage Inhibition and Toxin/Antitoxin Systems

Additional evidence of the role of TA systems for phage inhibition was provided 8 years later when it was shown the chromosomal type II MazF/MazE system inhibits phage P1 (Hazan and Engelberg-Kulka, 2004). Critically, *mazEF* deletions produced more P1 phage; hence, the phenotype of phage exclusion was verified without overproducing this TA system (Hazan and Engelberg-Kulka, 2004). Also, the type II RnlA/RnlB system inhibits T4 phage in *E. coli* (Koga et al., 2011).

In addition, 13 years after the discovery of phage inhibition by Hok/sok, the type III ToxN/ToxI system from plasmid pECA1039 of phytopathogen *Erwinia carotovora* was found to inhibit phage φA2 and φM1 when produced from a plasmid (Fineran et al., 2009), and 18 years later, the well-known abortive infection AbiEii/AbiEi system from plasmid pNP40 that inhibits the 936 phage family was suggested to be a type IV TA system (Dy et al., 2014). Hence, phage inhibition has been shown to be an important physiological role in types I, II, III, and IV TA systems.

Notably, in all TA systems tested for phage inhibition, there is no evidence of cell death during TA system activation under physiological conditions of toxin production, that is, via native toxin production levels (Song and Wood, 2018). However, it is somewhat difficult to differentiate possible activation of a toxin during phage invasion that leads to cell killing and killing from the phage itself, except that if the toxin kills the cell, phage progeny would be reduced. Similarly, there is no evidence of cell death under physiological conditions of toxin production for plasmid stabilization. Hence, phrases like “post-segregational killing” and “programmed cell death” should be avoided because activation of toxins of TA systems serves to reduce metabolism, not kill cells (based on the evidence to date) (Song and Wood, 2018).

## Phages Evolve Resistance to Phage Inhibition Systems

Phages and bacteria co-evolve (Stern and Sorek, 2011) to the extent that phages can be captured and utilized for the benefit of the host (Wang et al., 2009, 2010; Lee et al., 2018; Song et al., 2019). Hence, phages have developed means to thwart host anti-phage mechanisms. For example, phages have evolved myriad ways to undermine both restriction/modification (Stern and Sorek, 2011) and CRISPR-Cas (Bondy-Denomy et al., 2013; Rauch et al., 2017) systems. Therefore, if TA systems are *bona fide* phage inhibition systems, there should be examples of phage systems that undermine host phage exclusion mechanisms. Critically, to thwart bacterial phage inhibition systems, phages now have been identified that include antitoxins in their genome to inhibit host toxins; for example, T4 phage carries the Dmd antitoxin that inactivates both the RnlA/RnlB and LsoA/IsoB type II TA systems of *E. coli* O157:H7 (Otsuka and Yonesaki, 2012). T4 phage Dmd inactivates toxin RnlA by direct binding (Wei et al., 2016). Similarly, the mycobacterium phage Ibhubesi encodes a homolog of McbA, the antitoxin of the MbcT–MbcA TA system of *Mycobacterium tuberculosis* (Freire et al., 2019).

As additional evidence of phage evolving resistance to host TA systems utilized for phage inhibition, T7 phage produces the protease inhibitor Gp4.5 to prevent activation of host TA systems by inhibiting Lon protease, which is used by many TA systems to degrade antitoxins (Sberro et al., 2013). Hence, phage inhibition is an important physiological role of TA systems because four different types of TA systems inhibit phage and because phages have evolved defenses against TA systems.

In an additional role related to the co-evolution of phage and TA systems, whole, active TA systems have now been shown to be incorporated into phage genomes and used as regulatory units. Specifically, the PfiT/PfiA TA system is used by Pf4 filamentous phage of *Pseudomonas aeruginosa* to control phage production (Li et al., 2020).

## Malleable Toxin/Antitoxin Systems Evolved Into CRISPR-Cas Components

CRISPR-Cas is a prevalent anti-phage system present in about 40% of Bacteria and 90% of Archaea (Sorek et al., 2008). The first link between TA systems and CRISPR-Cas was based on bioinformatics and linked Cas proteins to toxin VapD based on protein sequence homology (Makarova et al., 2006). When we discovered the GhoT/GhoS type V TA system, the crystal structure of antitoxin GhoS linked it to Cas2 proteins of CRISPR-Cas, so another link between the two phage defense systems was established based on conservation of structure (Wang et al., 2012). TA systems are now considered the ancestors of Cas2 proteins (Makarova et al., 2020). This evolution of TA systems into CRISPR-Cas systems is supported by random mutagenesis studies, which have shown antitoxins like GhoS (type V) can be converted into a novel toxin ArT via only two amino acid substitutions, and antitoxins like MqsA (type II) and ToxI (type III) can be made to inhibit this *de novo* toxin (Soo et al., 2014). This concept of TA system malleability has been confirmed (Aakre et al., 2015).

## CRISPR-Cas Systems Mimic Toxin/Antitoxin Systems by Utilizing Growth Inhibition for Phage Inhibition

Critically, upon detecting invading DNA, the type III CRISPR-Cas system of *Streptococcus thermophilus* degrades not only the *invading* DNA but also non-specific *host* RNA through cyclic oligoadenylate signaling modification of Csm6 (Kazlauskiene et al., 2017). In addition, the *Staphylococcus epidermidis* subtype III-A CRISPR-Cas system causes general host growth arrest (but not cell death) through Csm6 when plasmids invade with inefficient DNA targets (Rostøl and Marraffini, 2019a). Hence, CRISPR-Cas in this species likely makes the host dormant to evade phage in a manner remarkably similar to TA systems, which reduce metabolism to limit phage propagation (Pecota and Wood, 1996). Further evidence of general host arrest as an anti-phage response has been found in the *Listeria seeligeri* type VI-A CRISPR-Cas system, which uses Cas13 to degrade host RNA upon phage invasion (Meeske et al., 2019); this CRISPR-Cas-induced host dormancy also protected neighboring cells from phage. Hence, inhibition of host metabolism by CRISPR-Cas systems is a common backup system to specific degradation of phage DNA, which mimics TA systems and their reduction in host growth to inhibit phage propagation. Moreover, it has been speculated that strains may utilize both CRISPR-Cas and TA systems for phage inhibition (Rostøl and Marraffini, 2019b).

## Perspectives

As summarized here, the most compelling arguments for phage inhibition as the primary physiological role of TA systems are (i) types I, II, III, and IV TA systems inhibit phage so this is a general mechanism; (ii) phages have evolved resistance to some bacterial TA phage exclusion systems to increase infection; and (iii) CRISPR-Cas systems, which are well-known phage inhibition strategies, mimic TA systems by reducing host metabolism to inhibit phage propagation.

Although the physiological role of phage inhibition by TA systems is well-established, to confirm phenotypes with new TA systems, we suggest that research should include experiments that show TA systems inhibit phage without over-producing the TA components; that is, TA loci should be deleted and the deletion strains tested for increased phage production. In this way, TA systems under physiological conditions will be further linked to phage inhibition.

Because phage attack is so prevalent, that is, there are 10-fold more phage than bacteria (Chibani-Chennoufi et al., 2004), perhaps the reason there are so many different TA systems in many bacterial genomes is not solely because of the various stresses bacteria encounter but also because different TA loci are utilized for different phages. Clearly, at this point in TA research, the answer to the question posed previously, “TA systems: why so many and what for” (Tsilibaris et al., 2007; Van Melderen, 2010), is that, rather than “devoid of any current physiological role” (Saavedra De Bast et al., 2008), they are used for the epic battle between bacterium and phage, that is, specifically they are primarily used for phage inhibition.

## Author Contributions

TW conceived and wrote the manuscript. SS helped writing the manuscript. Both authors contributed to the article and approved the submitted version.

## Conflict of Interest

The authors declare that the research was conducted in the absence of any commercial or financial relationships that could be construed as a potential conflict of interest.
